# A machine learning approach to identify optimal candidates for transarterial chemoembolization in unresectable HBV-related hepatocellular carcinoma complicated by first-branch portal vein tumor thrombus: a multicenter study

**DOI:** 10.3389/fonc.2026.1766607

**Published:** 2026-03-04

**Authors:** Suo Zhao, Qi Zhang, Yumeng Tang, Yichong Wang, Jiawei Song, Tenghui Han, Huadong Zhao, Yuheng Zhang, Chunzhi Yuan, Xiuqin Li, Jun Zhu, Lin Zhao

**Affiliations:** 1 The 987^th^ Hospital of the Joint Logistics Support Force of the People’s Liberation Army of China, Jibao, Shaanxi, China; 2 Department of General Surgery, The Southern Theater Air Force Hospital, Guangzhou, China; 3 Department of Digestive Surgery, The First Affiliated Hospital of Air Force Medical University, Xi’an, Shaanxi, China; 4 Department of Breast Cancer Center, West China Second University Hospital, Sichuan University, Chengdu, Sichuan, China; 5 Department of Neurology, Airborne Army Hospital, Wuhan, China; 6 Department of General Surgery, Tangdu Hospital, Air Force Medical University, Xi’an, China; 7 Department of Orthopedics, Western Theater Air Force Hospital, Chengdu, China

**Keywords:** first-branch portal vein tumor thrombus, HBV-related hepatocellular carcinoma, machine learning, propensity score matching, random survival forest, transarterial chemoembolization

## Abstract

**Background:**

Portal vein tumor thrombus (PVTT) is a critical factor influencing prognosis and treatment allocation for Hepatitis B virus (HBV)-related hepatocellular carcinoma (HCC). However, accurately selecting patients with unresectable HCC and first-order branch PVTT (PVTT1) who would benefit from transarterial chemoembolization (TACE) remains a significant clinical challenge. This study aimed to leverage machine learning to address this issue.

**Methods:**

We conducted a large-scale, retrospective multicenter study utilizing data from 15 tertiary hospitals in China (2012-2021). A Random Survival Forest (RSF) model was constructed to identify key prognostic variables and stratify risk among TACE-treated PVTT1 patients. Model performance was evaluated using the Area Under the Receiver Operating Characteristic Curve (AUC). To mitigate selection bias and confounding factors for survival comparisons, Propensity Score Matching (PSM) was performed.

**Results:**

Of the 3,948 patients enrolled, 763 constituted the TACE-PVTT1 group. The RSF model exhibited robust predictive accuracy for this group, identifying tumor size, tumor number, AST, INR, and age as the top five clinical predictors. Patients in the bottom risk-score tertile were classified as low-risk. Notably, the overall survival (OS) of this low-risk TACE-PVTT1 group was not significantly different from that of the 3,073 TACE-PVTT0 patients (P = 0.19), a finding that was maintained after PSM (P = 0.54). A multivariate Cox analysis confirmed that in this low-risk context, PVTT1 status was not a significant prognostic factor (P = 0.08575). Additionally, TACE conferred a significant survival advantage over sorafenib in patients with PVTT1.

**Conclusion:**

The integrated application of RSF and PSM can effectively identify low-risk candidates for TACE among patients with unresectable HCC and PVTT1. Our findings provide strong evidence that for this carefully selected patient subgroup, TACE offers survival outcomes comparable to those for patients without PVTT, highlighting the clinical utility of machine learning in guiding treatment decisions for this challenging disease.

## 1 Introduction

Hepatocellular carcinoma (HCC), classified as the fifth most common malignancy and the second leading cause of cancer-related deaths worldwide, represents a significant global health burden (1). Portal vein tumor thrombosis (PVTT), a dreadful complication, is indicative of advanced disease and poor patient outcome (2, 3). The incidence of PVTT ranges from 44% to 66.2%, and the median survival time for patients without any treatment is only 2.7 months (4, 5). Currently, there are numerous classification methods for PVTT based on the extent and scope of portal vein invasion, with the most prevalent in China being Cheng’s classification (6, 7).

Nevertheless, the optimal therapeutic strategy for HCC patients with PVTT remains to be elucidated, with ongoing debates about the most effective approaches. In Cheng’s classification, Type I PVTT (PVTT1) indicates the tumor thrombus invades the segmental portal vein or above. For this condition, both Transarterial chemoembolization (TACE) and sorafenib are established options for treatment (8, 9). Several studies showed that TACE may offer better overall survival (OS) outcomes to sorafenib, a first-line systemic therapy, suggesting a potential need to update the treatment strategy in HCC with PVTT1 management (10–12). However, no studies to date have identified the specific PVTT1 patient subset that stands to benefit from TACE therapy. Therefore, it warrants identifying and selecting appropriate candidates with PVTT1 for TACE represents an imperative and pressing challenge within clinical research.

Traditionally, the Cox regression proportional hazard analysis has been standard for prognostic evaluation by identifying the predicting factors (13). However, traditional methods struggle with the complexities of large datasets, while machine learning complements them for identifying suitable candidates (14). As a popular machine learning method, the Random Survival Forests (RSF) model can handle high-dimensional data and explore non-linear relationships between covariates and outcomes (15, 16). Additionally, Propensity Score Matching (PSM) used in observational studies matches patients with similar profiles to control for confounding, ensuring unbiased treatment effect estimates (17).

Our study aims to identify potential candidates for TACE for HCC patients with PVTT1 to improve their prognosis and foster precision therapy using the RSF algorithm and multivariate Cox and PSM analyses.

## 2 Patients and methods

### 2.1 Study design

A retrospective study was conducted on a multicenter database, comprising data from fifteen Chinese tertiary hospitals from January 2012 to December 2021. The patients were categorized into TACE-PVTT0, TACE-PVTT1, and sorafenib-PVTT1 cohorts: (1) TACE-PVTT0, consisting of HCC patients without any portal vein involvement who underwent transarterial chemoembolization (TACE); (2) TACE-PVTT1, comprising patients with Type I PVTT treated with TACE; and (3) sorafenib-PVTT1, including patients with Type I PVTT who received sorafenib monotherapy. The objective was to identify the best group of patients for TACE treatment for HCC within the TACE-PVTT1 group. An RSF model was consequently created for the TACE-PVTT1 group, with 85% of the data set allocated to the training set for model construction, while the remaining 15% was reserved for validation. The mortality risk scores predicted by the RSF model were used to stratify patients into three risk tiers (low, intermediate, and high). Using the tertile partitioning method, we defined the cutoff values at the 33.3rd and 66.7th percentiles of the risk score distribution in the training cohort.

The primary objective was to identify a low-risk subgroup among patients with PVTT1 who optimal candidates for TACE would be. Validation cohorts were used to validate and assess the model’s reliability. TACE-PVTT0 and sorafenib-PVTT1 cohorts were utilized to compare with low-risk TACE-PVTT1, attempting to demonstrate that our selected patients achieved results close to those of the PVTT0 patients who underwent TACE and significantly outperformed sorafenib-PVTT1.

**Figure 1:**
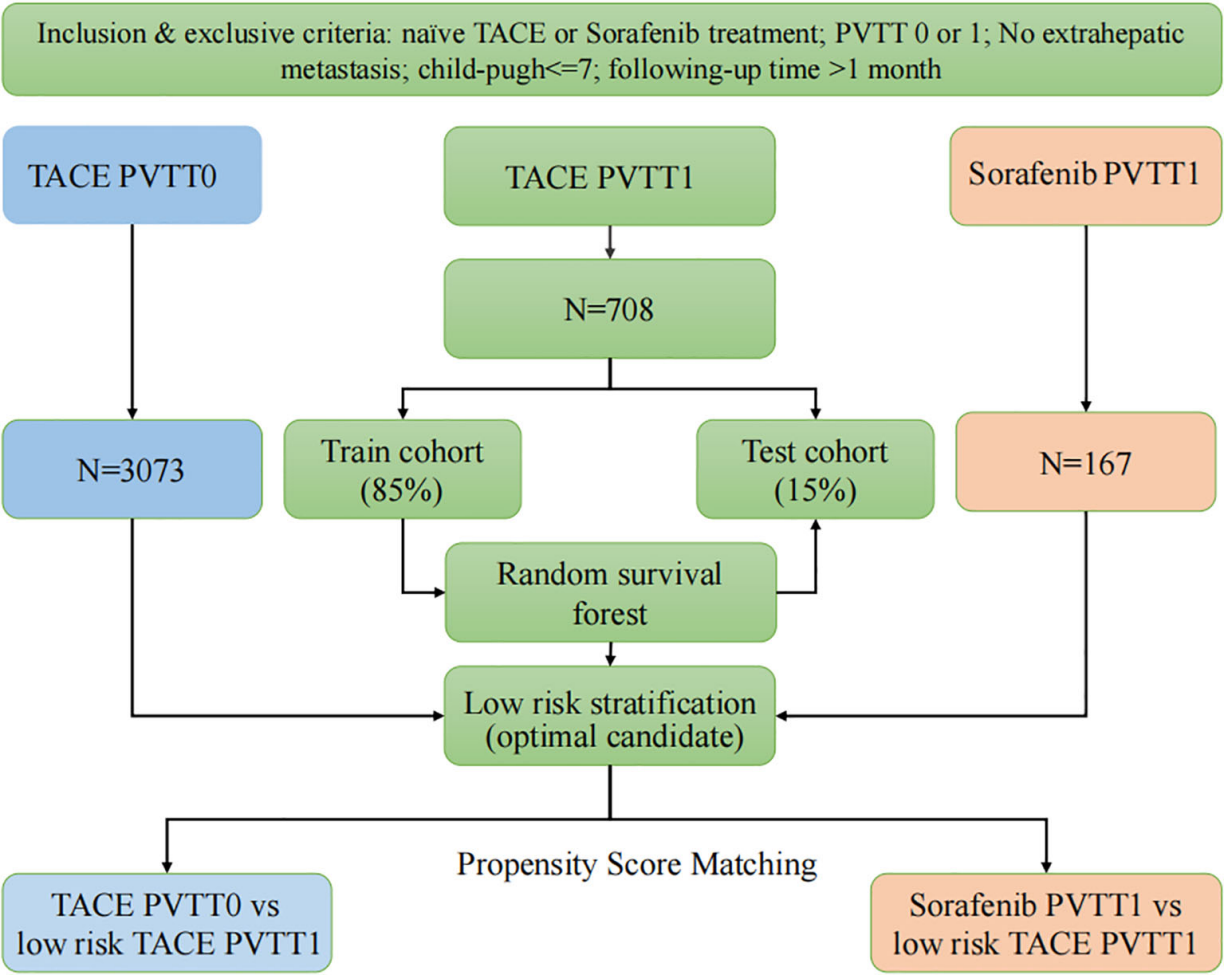
The flow chart of the study. PVTT0, without Portal vein tumor thrombus; PVTT1, first-order branch PVTT; low-risk TACE PVTT1, patients in the bottom tertile of the random survival forest model-derived risk scores.

Finally, to further verify the positive survival outcome based on the reduction of selection bias, the low-risk TACE-PVTT1 cohort was compared against the TACE-PVTT0 and sorafenib-PVTT1 cohorts through the integration of PSM and Cox regression analysis (**Figure 1**).

### 2.2 Data collection

A review of medical records was conducted to ascertain the clinical characteristics of patients undergone different treatments, and a total of 17 clinical parameters were collected. Tumor burden is an important factor in a range of variables, including the size and number of tumors. When collecting tumor size data, we recorded the maximum diameter of individual tumors.

We employed Cheng’s classification for the stratification of PVTT in HCC patients for its comprehensive consideration of the clinical characteristics and treatment practices specific to HCC patients. Chinese Cheng’s classification defines Type I PVTT based on the medical imaging results: the tumor thrombus invades the segmental portal vein or above. If the postoperative pathological result shows that the tumor thrombus is confined to microvascular, it is classified as type I (6, 18).

### 2.3 Population

HCC Patients were diagnosed according to the American Association for the Study of Liver Diseases/European Association for the Study of the Liver guidelines.

The patients were incorporated based on the following inclusion criteria: (1) diagnosed by HBV-related HCC; (2) patients with Type I PVTT (tumor thrombus limited to segmental or sectoral branches of the portal vein) who were treated by TACE or sorafenib; (3) patients without PVTT treated by TACE; (4) Child-Pugh class A or B7 (score ≤ 7); (5) Eastern Cooperative Oncology Group (ECOG) performance status of 0, 1 or 2.

The exclusion criteria were: (1) irreversible coagulopathy; (2) tumor thrombus extending into the first-order branches (Type II), the main portal vein trunk (Type III), or the superior mesenteric vein (Type IV); (3) severe infection or concurrent active hepatitis that cannot be effectively controlled; (4) diffuse tumor infiltration or extensive distant metastasis; (5) the proportion of tumor occupying the total liver size is ≥70% (not an absolute contraindication); (6) significant reduction in peripheral blood white blood cells and platelets, with white blood cells <3×10^9^/L and platelets <50×10^9^/L (not an absolute contraindication); (7) severe renal dysfunction: serum creatinine level of 176.8 μmol/L or creatinine clearance rate <30 mL/min.

The study was approved by the Ethics Committee of the fifteen hospitals. Informed consent was obtained from all patients before collection of data.

### 2.4 Treatment procedures

During the TACE procedure, a vascular catheter was inserted selectively into the tumor-feeding artery with an injection containing a mixture of doxorubicin (10–50mg) and lipiodol (2–20 ml), cisplatin (10–110 mg), epirubicin (10–50 mg), and oxaliplatin (100–200 mg), which were selected according to the practice of each center. Subsequently, either gelatin sponge or polyvinyl alcohol foam (PVA) particles were introduced, and embolization was monitored until angiography observed reduced blood flow in the tumor arteries. Repeat TACE was allowed when a residual viable tumor or a new lesion was identified in a patient with normal liver function.

Sorafenib was administered orally at a dosage of 400mg twice daily. The treatment should be continued without interruption, ceasing only in the event of disease progression or intolerable side effects. The dose modifications of sorafenib would be individualized based on each patient’s tolerability and toxicity profile. Patients are required to visit the outpatient clinic every 3 or 4 weeks for ongoing assessments of safety and therapeutic response. In instances of drug-related grade 3 or 4 toxicities, sorafenib dosage would be appropriately reduced to mitigate adverse effects while maintaining therapeutic efficacy. This approach ensures patient safety without compromising the potential benefits of treatment.

### 2.5 Follow-up

All patients were followed up one month after treatment with TACE or sorafenib therapy, then at 3-month intervals for the first year and every 6–12 months after that. In clinical practice, the frequency of follow-up appointments is tailored based on the patient’s baseline characteristics and the response to the most recent treatment.

OS was defined as the interval from initial TACE or sorafenib therapy to all-cause death. Patients who survived or lost to follow-up at the last follow-up date were censored.

### 2.6 Random survival forest

RSF is an ensemble machine learning method derived from the random forest algorithm, adapted to handle survival data, which may be censored. The method is applied to measure the minimal depth of a variable and rank the variable importance (VIMP) according to building multiple survival trees from bootstrap samples of the data. Under the assumption of minimal depth, the variates exerting a significant influence on predictive outcomes are predominantly those that consistently partition nodes close to the root. This occurs because these variables are responsible for segmenting the most expansive sample of the population. For VIMP, it quantifies the influence each predictor variable has on the outcome in a predictive model, thereby identifying the most impactful features within the dataset. We randomly selected a subset comprising 85% of the patients from the TACE-PVTT1 group to establish our training cohort. Within the training cohort, we employed RSF to ascertain the most influential variables contributing to the construction of decision trees which enables our subsequent work of model optimization. Linear predictive scores obtained by aggregating the predicted values of multiple decision trees inside the model were the indicators we used to quantify the results.

### 2.7 Propensity score matching

The PSM method was used to reduce the effect of selection bias and adjust for potential confounding factors, such as clinical stage, tumor size, and tumor number. Propensity scores were derived by fitting a logistic regression model based on age, gender, etiology, tumor size, therapy, AFP, ALB, TBIL, AST, ALT, PLT, INR, BUN, Cr, WBC, and HGB levels. The two groups were matched with a caliper width of 0.02, and 1:1 nearest neighbors matching without replacement was performed by the ‘MatchIt’ package in R software. This study used the PSM method to match patients with TACE-PVTT1 patients to those receiving sorafenib-PVTT1. Likewise, PSM was also used to match TACE-PVTT1 patients to TACE-PVTT0 patients.

### 2.8 Statistical analysis

The categorical variables in patients’ baseline demographics and clinical characteristics were described using frequencies and proportions. Additionally, continuous variables were described using measures of dispersion (median and interquartile range). The receiver operating characteristic Curve (ROC) and area under curve (AUC) were used to evaluate the predictive effectiveness of the model. K-M survival curves were applied to evaluate the probability of survival for different treatment groups, and hazard ratios (HR) and their 95% confidence intervals (CI) were calculated. The multivariate Cox regression analyses, applied to adjust the prognostic factors, were summarized by delineating the coefficient, HR, 95% CI, and corresponding P value for each covariable.

The RSF model was fitted using the “randomForestSRC” R package to predict the OS of patients based on the selected factors and the optimal hyperparameter combination. An “rms” R package was also used to develop a Cox regression analysis model. Two-tailed P-values < 0.05 were considered as statistically significant.

## 3 Results

### 3.1 Patient baseline characteristics

A total of 3948 patients with HCC were enrolled, including 3073 TACE-PVTT0, 763 patients with TACE-PVTT1, and 167 patients with sorafenib-PVTT1.

The baseline demographics and clinical characteristics of patients are shown in **Table 1**. Compared to the other two cohorts, patients in the TACE-PVTT0 group exhibited a significantly lower tumor burden, characterized by fewer tumor numbers and smaller tumor sizes. Additionally, the TACE-PVTT0 group demonstrated a lower incidence of elevated AFP levels (36.9% > 400 ng/mL) compared to the TACE-PVTT1 and sorafenib-PVTT1 groups (61.3% and 65.3% > 400 ng/mL, respectively). Furthermore, the age structure of the TACE-PVTT0 group is predominantly younger, hinting at a more advantageous demographic profile. In contrast, the sorafenib-PVTT1 group was identified as having the most substantial tumor burden, with an increased tumor number and expansive tumor sizes, signaling a more progressed stage of the disease.

**Table 1:** Basic clinical parameters in TACE and systematic therapy with PVTT0/1.

Characteristics	TACE PVTT0 N=3073	TACE PVTT1 N=763	Sys PVTT1 N=167
Gender:			
Female	449 (14.6%)	99 (13.0%)	27 (16.2%)
Male	2624 (85.4%)	664 (87.0%)	140 (83.8%)
Age:			
<60	1764 (57.4%)	544 (71.3%)	124 (74.3%)
≥60	1309 (42.6%)	219 (28.7%)	43 (25.7%)
Etiology:			
HBV	2711 (88.2%)	712 (93.3%)	154 (92.2%)
HCV	68 (2.21%)	3 (0.39%)	3 (1.80%)
HBV+HCV	19 (0.62%)	4 (0.52%)	0 (0.00%)
other	275 (8.95%)	44 (5.77%)	10 (5.99%)
ECOG:			
0	2064 (67.2%)	277 (36.3%)	49 (29.3%)
1	1000 (32.5%)	485 (63.6%)	118 (70.7%)
2	9 (0.29%)	1 (0.13%)	0 (0.00%)
Tumor number:			
1	1575 (51.3%)	337 (44.2%)	98 (58.7%)
2	783 (25.5%)	141 (18.5%)	38 (22.8%)
≥3	715 (23.3%)	285 (37.4%)	31 (18.6%)
Tumor size	6.10 [3.80;9.80]	10.5 [7.60;13.2]	11.0 [8.45;13.9]
PVTT:			
0	3073 (100%)	763 (100%)	0 (0.00%)
1	0 (0.00%)	0 (0.00%)	167 (100%)
AFP:			
<400	1940 (63.1%)	295 (38.7%)	58 (34.7%)
≥400	1133 (36.9%)	468 (61.3%)	109 (65.3%)
ALB	39.1 [35.6;43.0]	38.7 [35.4;42.0]	39.2 [36.0;42.0]
TBIL	16.3 [11.9;23.0]	17.2 [13.0;24.2]	18.4 [12.4;24.0]
AST	45.0 [31.0;68.0]	58.0 [44.0;91.0]	63.0 [46.5;93.0]
ALT	40.0 [26.0;62.0]	37.0 [28.0;62.0]	42.0 [28.0;66.0]
PLT	130 [85.0;184]	146 [102;204]	140 [95.0;207]
INR	1.06 [1.00;1.15]	1.08 [1.01;1.15]	1.10 [1.02;1.17]
BUN	5.30 [4.24;6.40]	4.40 [3.60;5.50]	4.60 [3.90;5.68]
Cr	72.0 [62.0;83.0]	70.0 [60.0;80.0]	80.5 [69.2;93.0]
WBC	5.24 [4.00;6.86]	5.38 [4.52;7.07]	5.43 [4.25;6.82]
HGB	135 [122;148]	129 [121;141]	139 [122;154]

Among the TACE-PVTT0, TACE-PVTT1 and sorafenib-PVTT1 groups, the median OS was 27.1 months, 8.03 months, and 6.9 months, and the median follow-up period was 36.5 months, 27.5 months and 45.1 months respectively. Patients in the TACE-PVTT0 subclassification had a significantly better OS than the others (**Supplementary Figure 1**).

### 3.2 RSF modeling in TACE-PVTT1 cohort

**Figure 2:**
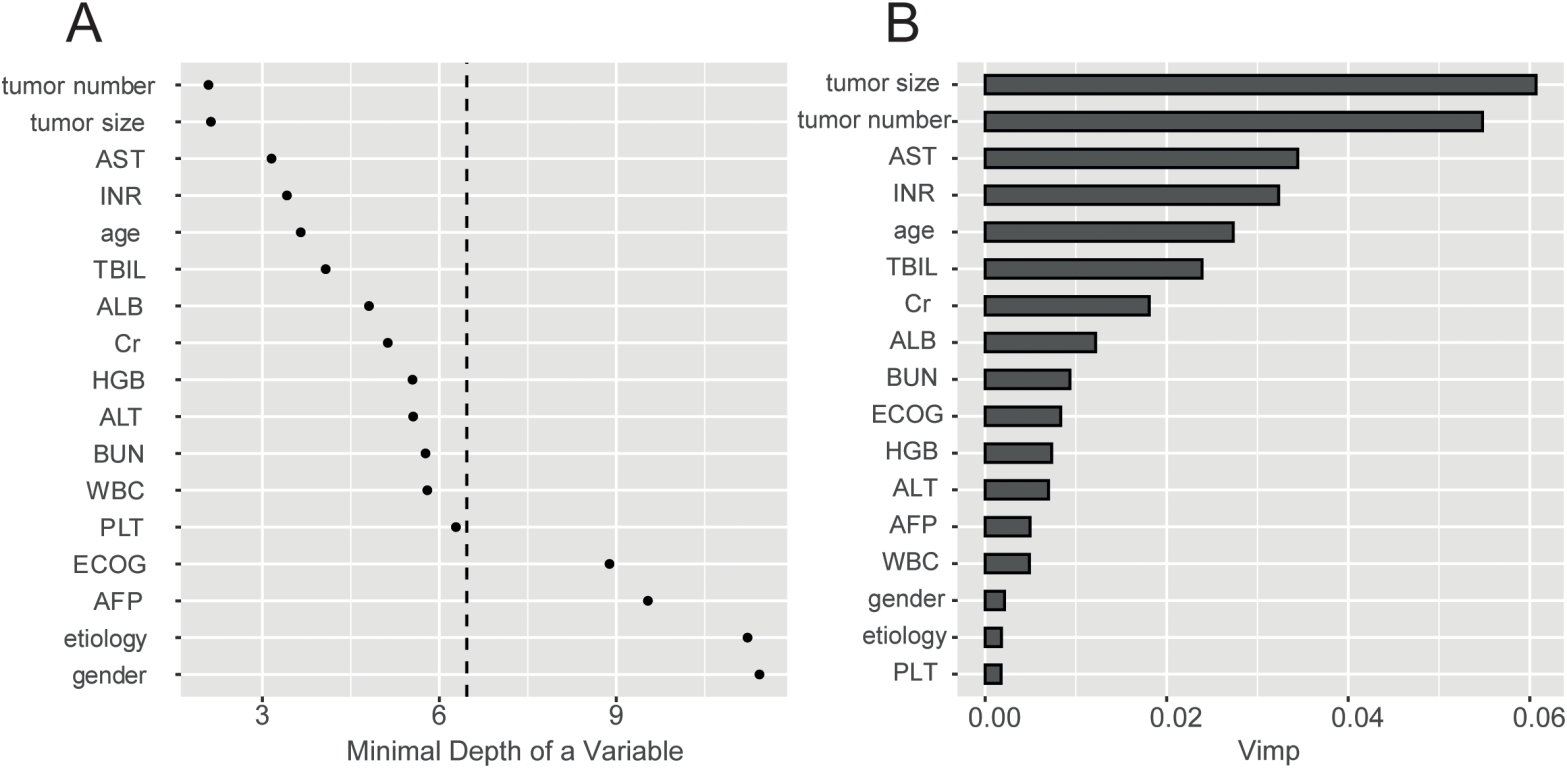
Dual-metric assessment of predictor importance from the RSF model. **(A)** Plot of minimal depth, which reflects the predictive directness of each variable. **(B)** Plot of permutation-based Variable Importance (VIMP), which measures the decrease in model accuracy when a variable’s values are randomized. RSF, Random Survival Forest; VIMP, Variable Importance; AST, Aspartate Aminotransferase; INR, International Normalized Ratio.

The model’s predictive capability typically improves when it is constructed using an expanded variable space. The training cohort consisted of 85% of patients with TACE-PVTT1 (n=649) in this study. A comprehensive collection of 17 covariates, encompassing demographic and clinical parameters, was collected and analyzed to identify potential predictive factors. We used the RSF model to calculate the minimal depth of a variable and rank the VIMP. It compares the minimum depth, and a lower minimal depth value indicates that this variable separates a large set of observations and therefore has a larger impact on forest prediction (**Figure 2A**). The VIMP of predicting patient survival probability is demonstrated in **Figure 2B**.

We randomly selected features for each sample and trained an RSF model with 1000 corresponding survival trees. The training cohort was established to build the model, and the validation cohort was established for model testing. The out-of-bag (OBB) error rate tended to be stable when the survival trees increased to a certain number (**Supplementary Figure 2A**). The minimal depth and VIMP methods were integrated to filter the variables better. It reveals that tumor size, tumor number, AST, INR, and age are the predominant factors significantly associated with predictive accuracy (**Supplementary Figure 2B**).

### 3.3 Validation and evaluation

**Figure 3:**
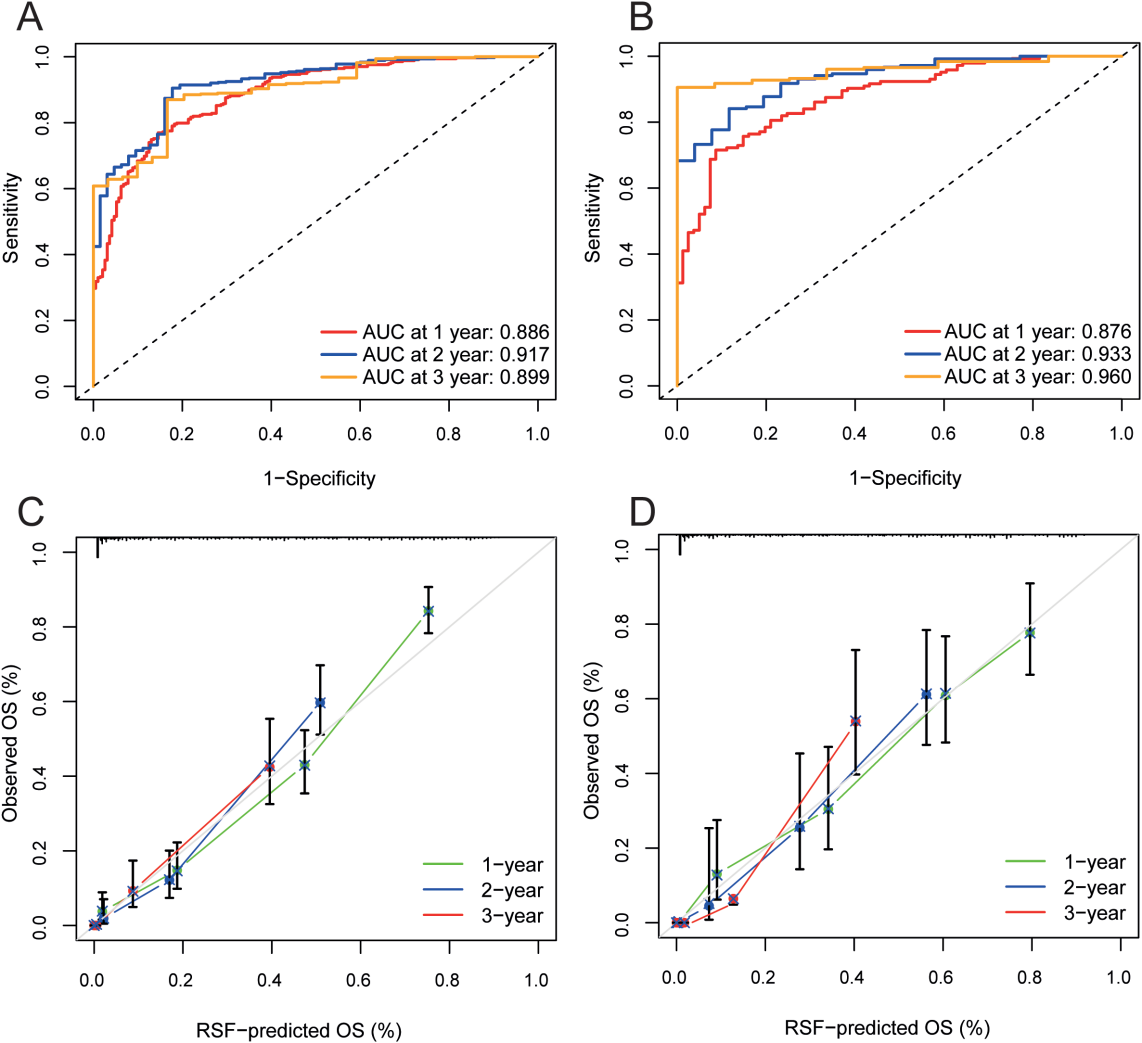
Evaluation of the RSF model in the training and validation cohort. **(A, B)** ROC curves of RSF model in the training **(A)** and validation cohort **(B)**. **(C, D)** Calibration curves of RSF model at 1-, 2-, 3-year in the training **(C)** and validation cohort **(D)**. RSF, Random Survival Forest; ROC, receiver operating characteristic curve.

The validation cohort consisted of 15% of those patients (n=114). Specifically, the AUC for the prediction of survival outcomes over the initial three-year period was 0.89, 0.92, and 0.89 in the training cohort, whereas that in the validation cohort reached a respective 0.88, 0.93, and 0.96, these results demonstrated an outstanding predictive capacity (**Figures 3A, B**). Furthermore, the calibration curves for these cohorts reveal a strong concordance between the model-predicted survival rates and the actual observed outcomes both the training cohort and the validation cohort showed excellent prediction ability, as presented in **Figures 3C, D**. The K-M survival curves showed significant differences when TACE PVTT1 group was stratified into low-risk, intermediate-risk and high-risk groups using trichotomy respectively (**Supplementary Figure 3A**). In addition to the survival probability, the age, ECOG, tumor size, AFP, ALB levels, and other clinical indicators of the low-risk group exhibited statistically significant disparities with medium- and high-risk groups (**Supplementary Table 1**, P<0.05). Patients were categorized into low, intermediate, and high risk using linear predictive scores, with ROC curves indicating survival probabilities at 1-, 2-, and 3- years, achieving AUCs of 0.84, 0.85, and 0.84, respectively. (**Supplementary Figure 3B**).

### 3.4 Development and validation of a simplified visualization tool

To enhance the interpretability and clinical applicability of our RSF model, we derived a simplified Nomogram based on the four most influential predictors: age, tumor number, INR, and tumor size (**Supplementary Figure 4A**). Each predictor was assigned a weighted score, and the total score was used to estimate the 1-, 2-, and 3-year survival rates. The calibration curves for the nomogram showed a high degree of consistency between the predicted and observed survival probabilities, indicating robust predictive performance and clinical reliability (**Supplementary Figure 4B**).

The total cohort was partitioned into training and internal testing sets using an 85:15 ratio. To ensure that this specific allocation did not bias the results, a sensitivity analysis was performed using a standard 80:20 split, which yielded consistent predictive performance (**Supplementary Figure 5**, AUC range: 0.884–0.945), thereby confirming the robustness of the model across different data-partitioning strategies.

### 3.5 TACE-PVTT0 vs low-risk TACE-PVTT1

**Figure 4:**
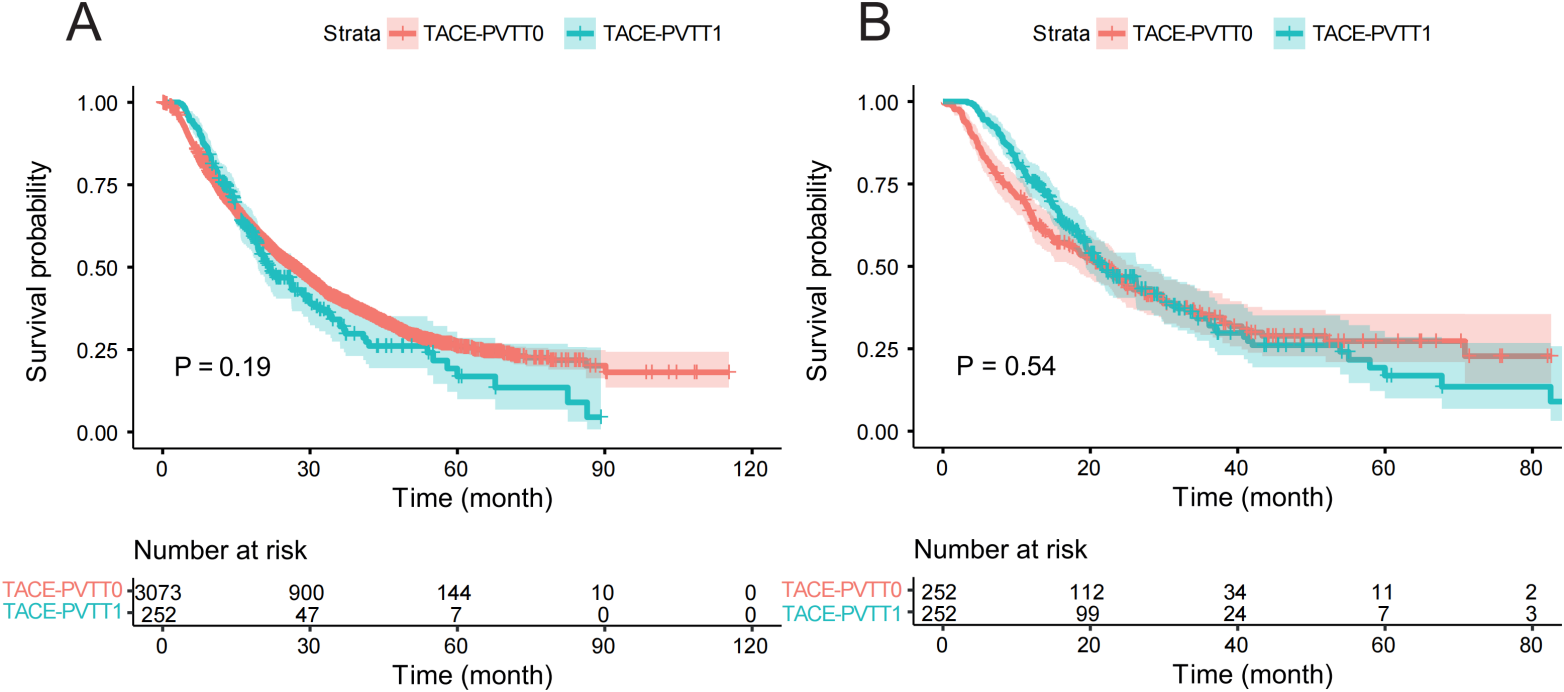
The survival curve of TACE-PVTT0 and low-risk TACE-PVTT1. The survival differences between TACE-PVTT0 and low-risk TACE-PVTT1 before PSM **(A)** and after PSM **(B)**. TACE-PVTT0, patients without PVTT and underwent TACE therapy, low-risk TACE PVTT1, patients in the bottom tertile of the RSF model-derived risk scores; PSM, Propensity Score Matching.

In addition, we compared the OS in TACE-PVTT0 and low-risk TACE-PVTT1 groups which showed no significance (P = 0.19, **Figure 4A**). However, the effect of selection bias and potential confounding factors still existed, and some significant differences in some covariables were not excluded. Significant differences in clinical and pathological covariates are observed between the two cohorts (**Supplementary Table 2**).

After performing PSM to minimize selection bias, a total of 252 matched pairs of patients were identified with no significant difference in any covariates between the TACE-PVTT0 and low-risk TACE-PVTT1 groups, and the consistency of OS of the two groups was further confirmed (P = 0.54, **Figure 4B**). **Supplementary Table 3** presents the baseline demographics and clinical characteristics between low-risk TACE-PVTT1 and TACE-PVTT0 groups following PSM. Consequently, patients categorized as low-risk group were deemed optimal candidates for TACE treatment in PVTT1.

To further evaluate the comparative efficacy of TACE-PVTT1 and low-risk TACE-PVTT0, we conducted a comprehensive multivariate Cox analysis. The results presented in **Table 2** showed that the presence or absence of PVTT did not exert a significant effect on prognosis (P = 0.08). Conversely, a cohort of covariates, including ECOG, AFP levels, tumor number, and tumor size, demonstrated a substantial correlation with survival risks post-TACE treatment, achieving statistical significance (P < 0.05). However, these differences were eradicated after PSM (**Supplementary Table 6**).

**Table 2:** Multivariable Cox analysis of TACE PVTT0 with low risk TACE PVTT1.

Characteristics	Coefficient	HR	Lower 95%CI	Upper 95%CI	P value
PVTT (no as ref)	-0.1512	0.8597	0.7234	1.0215	0.08575
Gender (Female as ref)	-0.03559	0.965	0.8443	1.103	0.6016
Age (<60y as ref)	0.0017	1.0017	0.9976	1.0059	0.4121
Etiology (HBV as ref)					
HCV	-0.0255	0.9748	0.7070	1.3442	0.8764
HBV+HCV	0.3991	1.4904	0.9090	2.4437	0.1131
other	-0.1034	0.9018	0.7647	1.0634	0.2191
ECOG (0 as ref)					
1	0.2567	1.2927	1.1742	1.4232	<0.0001
2	0.3883	1.7009	0.8060	3.5893	0.1633
AFP (<400 as ref)	0.3086	1.3614	1.2385	1.4966	<0.0001
Tumor number (1 as ref)					
2	0.2933	1.3408	1.1969	1.5019	<0.0001
≥3	0.5592	1.7493	1.565	1.9552	0.0010
Tumor size	0.0919	1.0963	1.0829	1.1099	<0.0001
ALB	-0.0155	0.9846	0.9749	0.9944	0.0022
TBIL	0.0067	1.0067	1.0025	1.011	0.0016
AST	-0.0002	0.9998	0.9990	1.0006	0.583762
ALT	-0.0007	0.9993	0.9984	1.0002	0.1330
PLT	0.0002	1.0002	0.9997	1.0006	0.3987
INR	0.5903	1.8045	1.3072	2.4911	0.0003
BUN	-0.0049	0.9951	0.9830	1.0075	0.4379
Cr	0.0016	1.0016	0.9998	1.0034	0.0802
WBC	0.0007	1.0008	0.9997	1.0018	0.1749
HGB	0.0004	1.0004	0.998	1.0028	0.7530

### 3.6 sorafenbib-PVTT1 vs low-risk TACE-PVTT1

**Figure 5:**
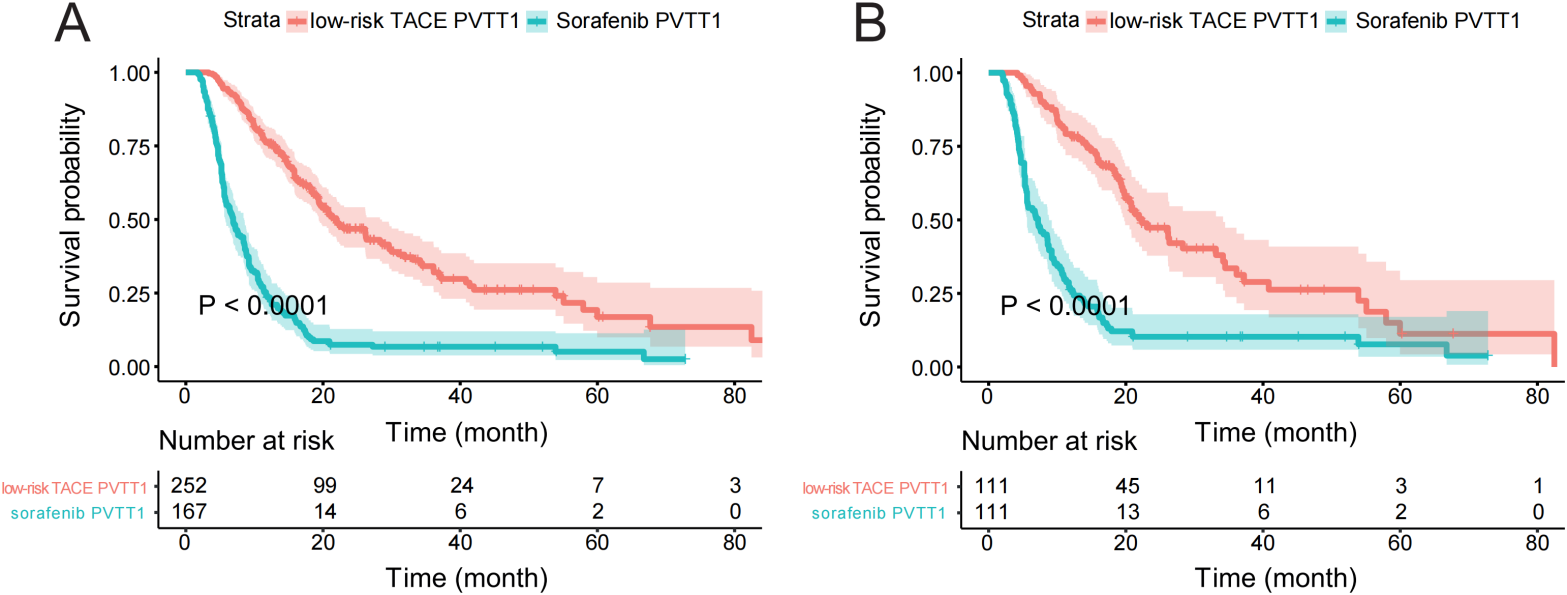
The survival curve of sorafenib-PVTT1 and low-risk TACE-PVTT1. The survival differences between sorafenib-PVTT1 and low-risk TACE-PVTT1 before PSM **(A)** and after PSM **(B)**. sorafenib-PVTT1, patients with PVTT1 and underwent sorafenib therapy, low-risk TACE PVTT1, patients in the bottom tertile of the RSF model-derived risk scores; PSM, Propensity Score Matching.

A comparative analysis between sorafenib-PVTT1 and low-risk TACE-PVTT1 groups was also conducted, revealing a markedly superior OS rate in the low-risk TACE-PVTT1 group as opposed to sorafenib therapy, both pre-and post-PSM, as illustrated in **Figure 5** (P < 0.05). These findings demonstrate that the model aids in the precise selection of PVTT1 patients who are more likely to benefit from TACE over sorafenib therapy. We provide evidence that the PSM method effectively adjusts for potential confounding factors, thereby minimizing irrelevant impacts of certain covariates on the comparison (**Supplementary Tables 4**, **5**).

Moreover, we provided insights into the prognostic implications of TACE and sorafenib treatment approaches in the context of PVTT1 through a comprehensive multivariate Cox regression analysis, which showed that the choice of therapy had a significant influence on prognosis (P < 0.05), as detailed in **Table 3**. Notably, the HR for sorafenib therapy, with TACE as the reference category, is elevated to 3.4, which further escalates to 7.6 after PSM, as documented in **Supplementary Table 7**. This substantial increase underscores a significant difference in the efficacy of the two treatment modalities. Additionally, the remaining covariates did not exhibit statistically significant differences in their impact on prognosis. These results indicate the significant clinical relevance of our model, demonstrating its utility in guiding therapeutic decision-making and patient management.

**Table 3:** Multivariable Cox analysis of TACE and systemic therapy with PVTT1.

Characteristics	Coefficient	HR	Lower 95%CI	Upper 95%CI	P value
Therapy (TACE as ref)	1.2293	3.4187	2.5709	4.5462	<0.0001
Gender (Female as ref)	-0.2850	0.7520	0.5361	1.0548	0.0988
Age (<60y as ref)	-0.1213	0.8858	0.6740	1.1641	0.3844
Etiology (HBV as ref)					
HCV	0.3056	1.3575	0.2488	7.4066	0.7240
HBV+HCV	1.5962	4.9343	1.1497	21.1774	0.0317
other	-0.1089	0.8968	0.5719	1.4064	0.6352
ECOG (0 as ref)					
1	-0.0096	0.9904	0.7666	1.2796	0.9413
2	0.8135	2.2557	0.2908	17.4994	0.4364
AFP (<400 as ref)	-0.009115	0.9909	0.7617	1.2891	0.9459
Tumor number (1 as ref)					
2	-0.0751	0.9276	0.6684	1.2875	0.6534
3	-0.0929	0.9113	0.6644	1.2498	0.5642
Tumor size	0.0560	1.0576	1.0169	1.1	0.0051
ALB	0.0031	1.0031	0.9721	1.0351	0.8462
TBIL	0.0092	1.0092	0.9918	1.027	0.3002
AST	0.0006	1.0006	0.9969	1.0043	0.7476
ALT	-0.0048	0.9952	0.9905	0.9998	0.0431
PLT	0.0003	1.0003	0.9986	1.002	0.7026
INR	0.1733	1.1892	0.3348	4.224	0.7887
BUN	-0.0093	0.9907	0.9114	1.0769	0.8263
Cr	0.0035	1.0035	0.9965	1.0105	0.3283
WBC	0.0028	1.0028	0.9509	1.0576	0.9175
HGB	0.0034	1.0034	0.9955	1.0114	0.3974

## 4 Discussion

Our study presents a novel application of machine learning methods, especially the RSF model, to identify optimal candidates for TACE treatment among patients with HCC and first-order branch PVTT. The integration of these methods effectively predicted the prognosis of TACE treatment and described a subgroup of low-risk TACE-PVTT1 patients, suggesting that TACE is an ideal treatment option for this subgroup. Our RSF model successfully identified variables significantly associated with the prognosis of TACE treatment, including tumor size, tumor number, AST, INR, and age. The application of PSM further confirmed the non-significance in OS between low-risk TACE-PVTT1 and TACE-PVTT0 patients. In addition, survival outcomes for TACE-PVTT1 showed a significant improvement compared to sorafenib-PVTT1, suggesting that TACE is a viable option for the PVTT1 subset of patients.

In our study, a range of factors were identified that significantly impact survival outcomes. Based on the VIMP from our model and multivariate Cox analysis, the results underscore the significant predictive effect of tumor burden (number and size of lesions), AST, age, and AFP levels on the accuracy of survival predictions. These findings align with established studies, which have consistently demonstrated the prognostic relevance of these predictors in HCC (19–22). Song et al. indicate that larger tumor size and greater number significantly affect the prognosis of hepatocellular carcinoma, correlating with shorter survival and faster progression (19). Wang et al. also illustrated the contribution of tumor size and number to prognosis and provided a method for assessing tumor burden (23). Chen et al. indicated that AST is an independent factor associated with short-term survival rates (21). In this study, the AFP levels ranked high in the VIMP, possibly because AFP reveals the heterogeneity of the tumor, thereby having a greater impact on prognosis. The consistency of our results with prior research substantiates the reliability of these biomarkers in prognostic assessments and reinforces their utility in clinical decision-making for HCC patients. The previous study indicates AFP and tumor burden are related to the survival outcome (21, 24, 25). Similarly, the VIMP measure also demonstrated the significance of these variables concerning survival outcomes in our study. Recent research indicates that for patients with unresectable HCC undergoing systemic treatment, early clinical hepatic decompensation (CHD) is the strongest predictor of mortality, and the International Normalized Ratio (INR) correlates with the development of early CHD (26). Similarly, INR ranks high in our RSF model. This prominence may be attributed to the utilization of RSF, which excels in constructing multivariate models, and the non-linear relationships between INR and survival outcomes have been effectively captured.

Prior studies have explored the impact of PVTT on the prognosis of HCC patients. Xiang et al. compared the efficacy of TACE versus the best supportive care in patients with HCC and PVTT, potentially aiding in identifying patient characteristics most suitable for TACE treatment (27). Silva et al. assessed the efficacy of TACE with PVTT, potentially providing evidence for selecting patients who are candidates for TACE treatment (28). Zhang et al. indicated that liver resection is more appropriate for PVTT1, but the low-risk PVTT1 patients selected have achieved therapeutic effects close to those of PVTT0, offering a new perspective for clinical decision-making (29). However, few have utilized machine learning to identify potential candidates for TACE treatment, our study addresses this gap. The application of machine learning techniques, particularly the RSF model, enables the more precise identification of low-risk PVTT1 patients who are most likely to benefit from TACE treatment among a large patient population. Our results also demonstrate improved differentiation.

The therapeutic landscape for advanced HCC has undergone a paradigm shift, as the combination of molecular targeted therapy and immunotherapy has significantly improved patient prognosis (30). Recent landmark trials, such as the IMbrave150 and ORIENT-32 (31), demonstrate that immunotherapy-based regimens or the integration of TACE with systemic therapies provide superior survival outcomes compared to traditional monotherapies (32, 33).

However, this evolution does not imply that TACE has lost its clinical value; rather, TACE remains a mature cornerstone of multimodal treatment (34). Our RSF model, although derived from a retrospective cohort (2012–2021) lacking a dedicated “TACE plus systemic therapy” arm, serves as a foundational prognostic “scaffold.” The core variables identified—such as baseline tumor aggressiveness and hepatic reserve—are foundational prognostic anchors that persist across different therapeutic eras. Because the RSF algorithm excels at capturing non-linear interactions, it can be readily adapted to modern paradigms by incorporating “treatment modality” as a high-order categorical variable in future iterations.

In the current era of high-cost combination therapies, selecting optimal candidates for TACE-based interventions remains crucial. Our model facilitates precision oncology by identifying high-risk individuals who may require more aggressive, multi-agent protocols, thereby bridging the gap between traditional interventional oncology and modern systemic immunotherapy.

Despite the methodological innovation of our study, several limitations should be acknowledged. Firstly, as a retrospective study, there is a potential for unidentified confounding factors, although we employed methods such as PSM to mitigate known biases. Secondly, although our database comprises 1,570 patients from 15 tertiary hospitals, the validation was conducted using an internal split of the pooled data rather than a “true” external validation using a completely independent institution. While this pooled approach reflects real-world heterogeneity across various centers and our sensitivity analysis confirms the model’s robustness, the generalizability of our findings to independent cohorts and other ethnic populations remains to be further established in future prospective studies. Thirdly, the TACE protocols, including chemotherapeutic agents and embolic materials, were not standardized across the 15 centers over the 10-year period, which reflects real-world therapeutic heterogeneity but may introduce procedural bias. Finally, although the RSF model provides variable importance scores, machine learning methods are inherently less intuitive than traditional statistical methods. While we have developed a simplified nomogram to bridge this gap, further efforts to enhance the transparency and interpretability of such “black-box” models are still warranted to facilitate their clinical adoption.

In summary, our study harnessed the RSF algorithm and multivariate Cox analyses to identify optimal TACE candidates among HCC patients with PVTT1, advancing our pursuit of precision therapy and offering a pathway for improved patient outcomes. This novel approach may provide useful guidance and new perspectives for clinical decision-making.

## Data Availability

The original contributions presented in the study are included in the article/**Supplementary Material**. Further inquiries can be directed to the corresponding authors.
