# Supplementary material for: A machine learning approach to identify optimal candidates for transarterial chemoembolization in unresectable HBV-related hepatocellular carcinoma complicated by first-branch portal vein tumor thrombus: a multicenter study
Source: Front Oncol. 2026 Mar 4;16:1766607. doi: 10.3389/fonc.2026.1766607 (PMC12995787; doi:10.3389/fonc.2026.1766607)
Supplement: Supplementary file 1 [file DataSheet1.docx]

Supplementary materials

**Title: A Machine Learning Approach to Identify Optimal Candidates for Transarterial Chemoembolization in Unresectable HBV-related hepatocellular carcinoma complicated by First-Branch Portal Vein Tumor Thrombus: a multicenter study**

Figure S1: Survival analysis of TACE and sorafenib groups.

Figure S2: Performance and interpretation of the Random Survival Forest (RSF) model.

Figure S3: Performance of the RSF Model for Risk Stratification and Prediction.

Figure S4: Construction of nomogram and calibration curve.

Figure S5: Sensitivity analysis of the RSF model using an 80:20 training-to-testing partition ratio.

Note: RSF, Random Survival Forest; VIMP, Variable Importance; ROC, Receiver Operating Characteristic; AUC, Area Under the Curve; INR, International Normalized Ratio.

Table S1: Basic clinical characteristics of different risk of PVTT1 underwent TACE.

Table S2: Pre-PSM Comparison of TACE without PVTT and low risk PVTT1 underwent TACE

Table S3: Post-PSM Comparison of TACE without PVTT and low risk PVTT1 underwent TACE

Table S4: Pre-PSM Comparison of systematic therapy and TACE with PVTT1

Table S5: Post-PSM Comparison of systematic therapy and low risk TACE with PVTT1

Table S6: Multivariable Cox analysis of TACE PVTT0 with low risk TACE PVTT1 after PSM

Table S7: Multivariable COX analysis of TACE and systematic therapy with PVTT1 after PSM.


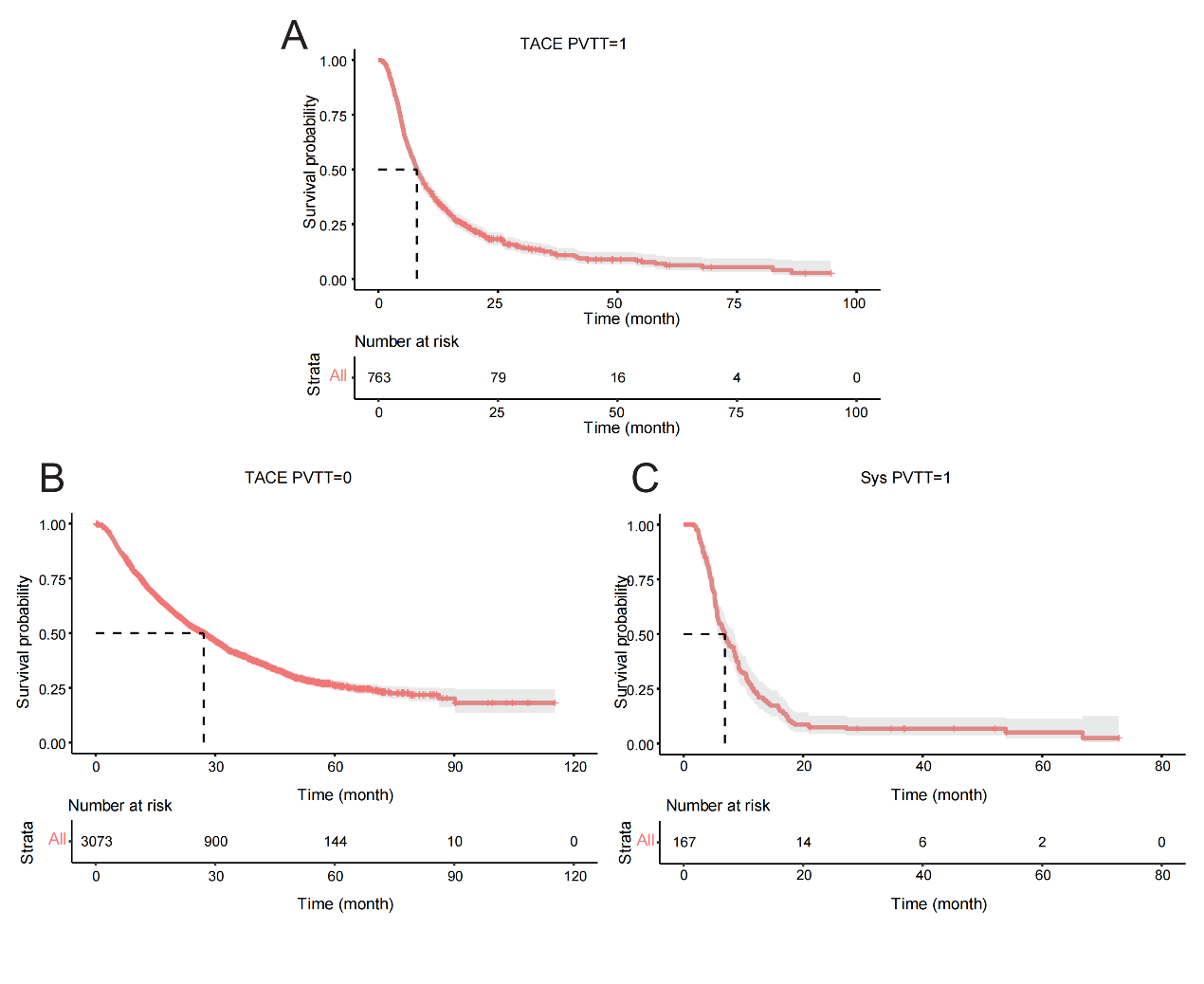
 Figure S1: Survival analysis of TACE and sorafenib groups.

Kaplan-Meier curves of patients with PVTT1 underwent TACE therapy (A), patients without PVTT underwent TACE therapy (B) and patients with PVTT1 underwent sorafenib systemic therapy (C).


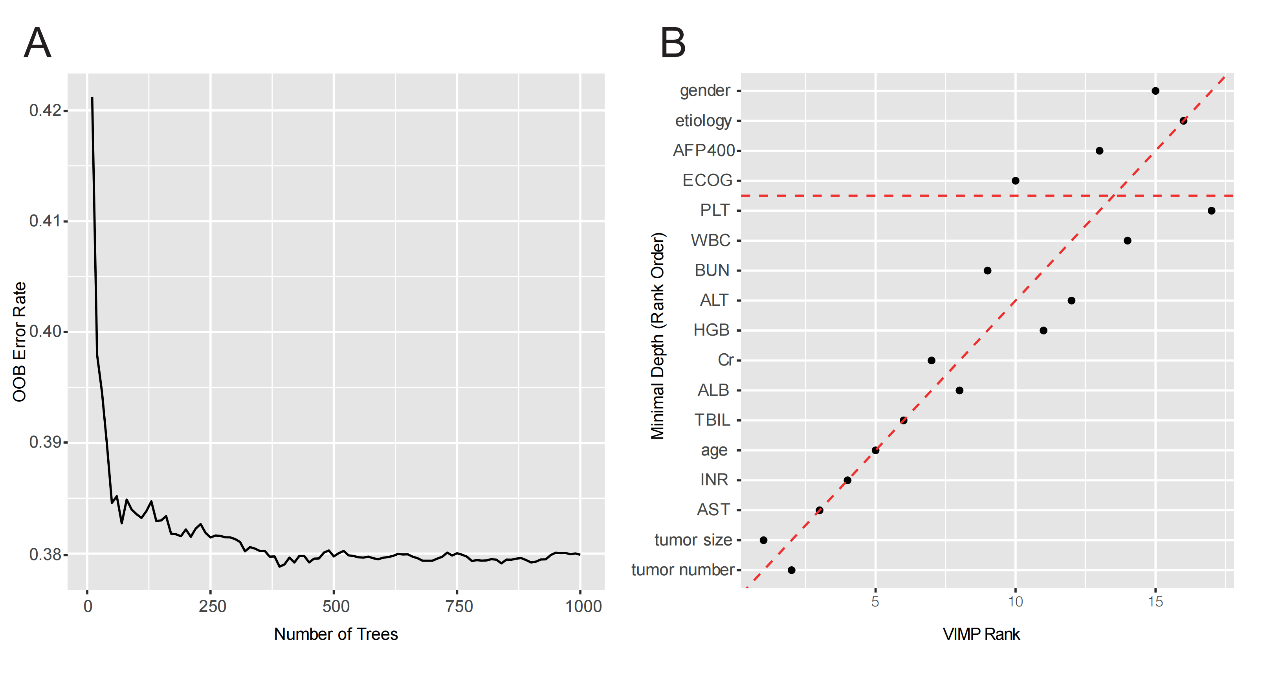


Figure S2 Performance and interpretation of the Random Survival Forest (RSF) model.

(A) The Out-of-Bag (OOB) error rate stabilized as the number of trees grew to 1000, indicating model convergence. (B) The top five most important clinical predictors for overall survival identified by the model were tumor size, tumor number, AST, INR, and age.


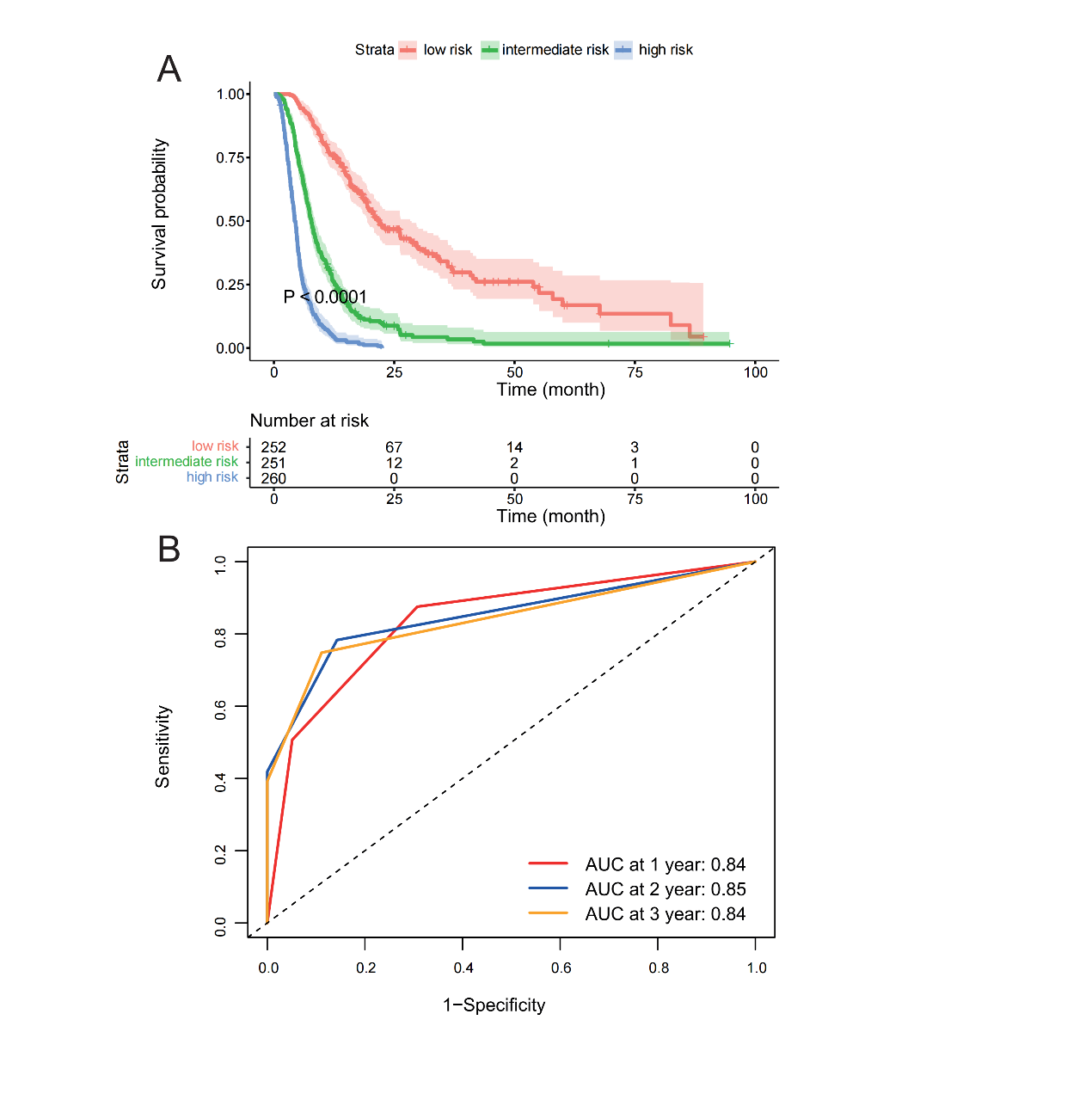


Figure S3 Performance of the RSF Model for Risk Stratification and Prediction.

(A) Kaplan-Meier curves for the low-, middle-, and high-risk groups defined by the RSF model (P < 0.0001). (B) Time-dependent ROC curves for the RSF model at 1-, 2- and 3- years, with corresponding AUC values.


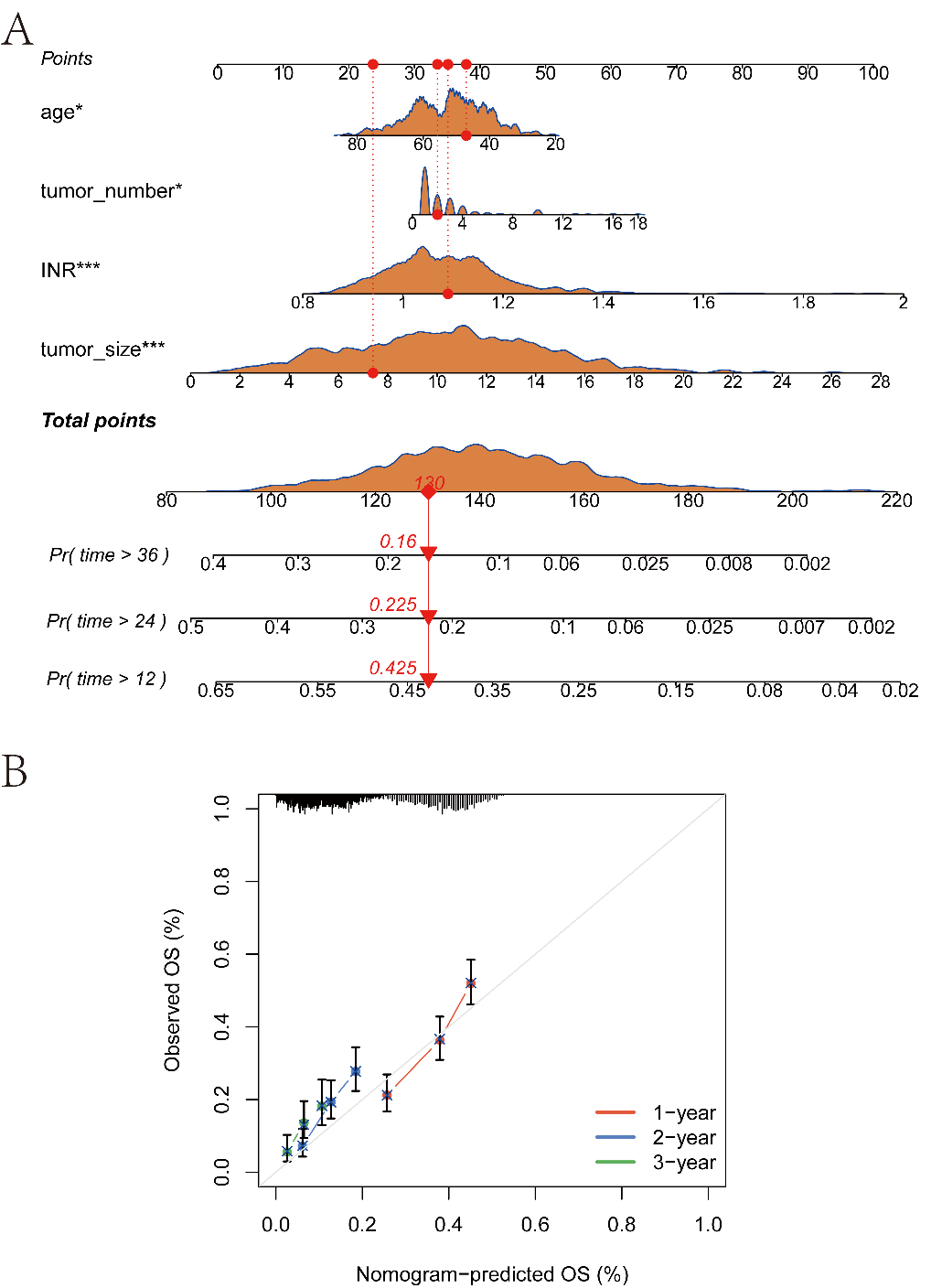


Figure S4: Construction of nomogram (A) and calibration curve (B)


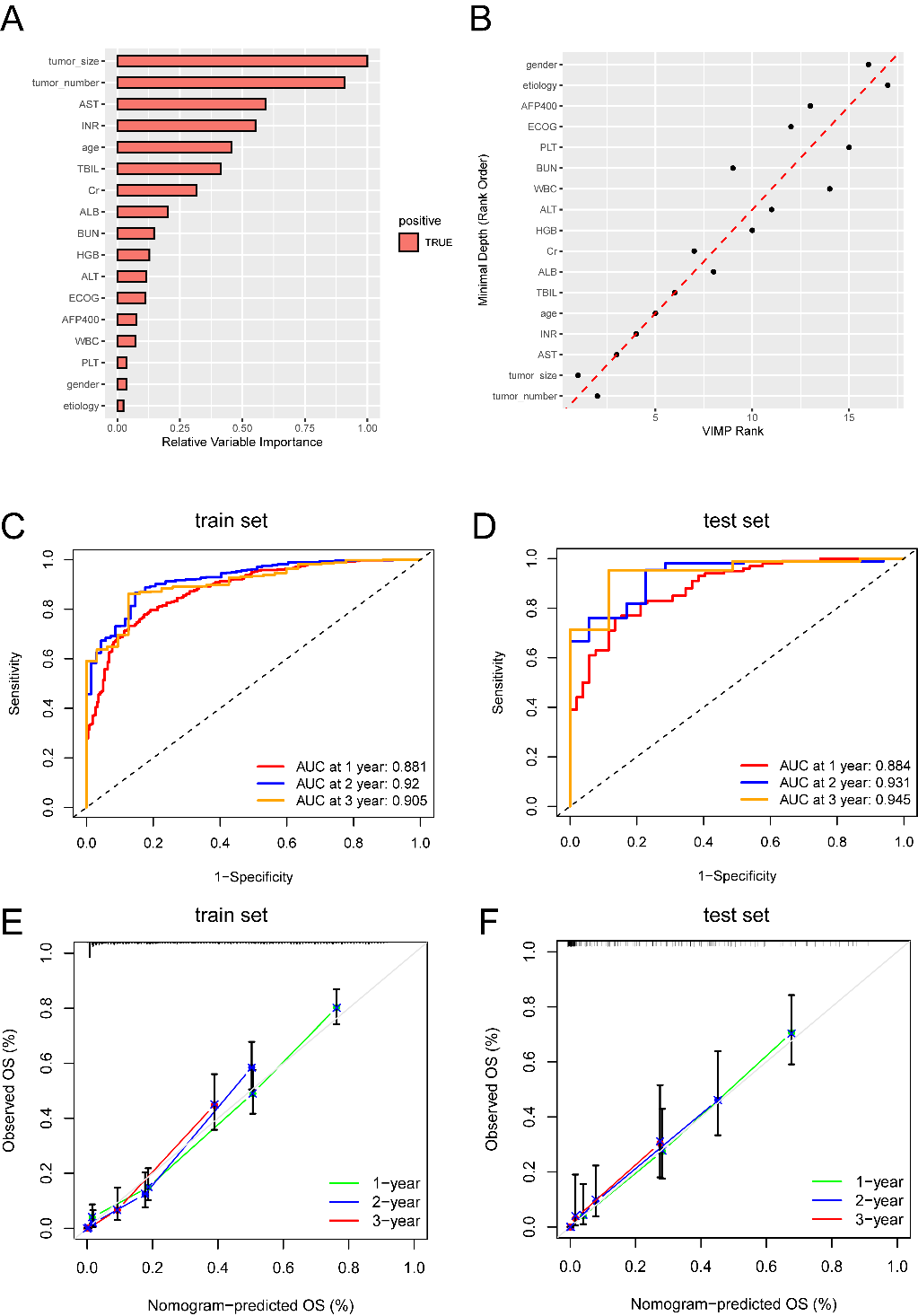


Figure S5. Sensitivity analysis of the RSF model using an 80:20 training-to-testing partition ratio.

(A) Relative variable importance of the predictive parameters, showing the contribution of each clinical feature to the model's performance.

(B) Ranking of variables based on the Variable Importance (VIMP) scores, confirming the stability of key predictors including age, AST, tumor number, INR, and tumor size.

(C-D) Time-dependent ROC curves for predicting 1-, 2-, and 3-year overall survival in the training cohort (C) and the internal validation cohort (D) under the 80:20 split. The AUC values at different time points demonstrate consistent discriminative power.

(E-F) Calibration plots for the training cohort (E) and internal validation cohort (F).

RSF, Random Survival Forest; VIMP, Variable Importance; ROC, Receiver Operating Characteristic; AUC, Area Under the Curve; INR, International Normalized Ratio.

Table S1: Basic clinical characteristics of different risk of PVTT1 underwent TACE.

| Characteristics | Low risk  N=252 | Intermediate risk  N=251 | High risk  N=260 | P value |
| --- | --- | --- | --- | --- |
| Gender: |  |  |  | 0.002 |
| Female | 48 (19.0%) | 28 (11.2%) | 23 (8.85%) |  |
| Male | 204 (81.0%) | 223 (88.8%) | 237 (91.2%) |  |
| Age: |  |  |  | <0.001 |
| <60 | 149 (59.1%) | 176 (70.1%) | 219 (84.2%) |  |
| ≥60 | 103 (40.9%) | 75 (29.9%) | 41 (15.8%) |  |
| Etiology: |  |  |  | 0.061 |
| HBV | 226 (89.7%) | 238 (94.8%) | 248 (95.4%) |  |
| HCV | 1 (0.40%) | 2 (0.80%) | 0 (0.00%) |  |
| HBV+HCV | 2 (0.79%) | 1 (0.40%) | 1 (0.38%) |  |
| other | 23 (9.13%) | 10 (3.98%) | 11 (4.23%) |  |
| ECOG: |  |  |  | <0.001 |
| 0 | 130 (51.6%) | 85 (33.9%) | 62 (23.8%) |  |
| 1 | 121 (48.0%) | 166 (66.1%) | 198 (76.2%) |  |
| 2 | 1 (0.40%) | 0 (0.00%) | 0 (0.00%) |  |
| Tumor number |  |  |  | 0.024 |
| 1 | 128 (50.8%) | 107 (42.6%) | 102 (39.2%) |  |
| 2 | 49 (19.4%) | 40 (15.9%) | 52 (20.0%) |  |
| ≥3 | 75 (29.8%) | 104 (41.4%) | 106 (40.8%) |  |
| Tumor size | 8.50 [5.30;10.7] | 10.9 [8.50;13.0] | 12.5 [9.38;15.1] | <0.001 |
| AFP: |  |  |  | <0.001 |
| <400 | 134 (53.2%) | 92 (36.7%) | 69 (26.5%) |  |
| ≥400 | 118 (46.8%) | 159 (63.3%) | 191 (73.5%) |  |
| ALB | 39.5 [37.2;43.0] | 38.2 [35.2;41.2] | 37.7 [34.7;41.4] | <0.001 |
| TBIL | 15.6 [11.9;19.3] | 16.3 [12.0;21.9] | 21.6 [16.4;28.2] | <0.001 |
| AST | 46.6 [35.0;60.2] | 56.0 [46.0;83.0] | 82.0 [58.0;128] | <0.001 |
| ALT | 35.0 [25.0;50.2] | 37.0 [27.5;61.0] | 40.0 [32.0;72.0] | <0.001 |
| PLT | 133 [94.0;202] | 163 [114;204] | 142 [103;208] | 0.009 |
| INR | 1.04 [0.97;1.12] | 1.07 [1.02;1.14] | 1.13 [1.04;1.20] | <0.001 |
| BUN | 4.60 [3.90;6.50] | 4.30 [3.75;5.50] | 4.30 [3.50;5.40] | 0.001 |
| Cr | 70.0 [58.0;80.0] | 70.0 [60.0;81.0] | 70.0 [61.0;79.0] | 0.708 |
| WBC | 5.13 [4.52;6.98] | 5.55 [4.52;7.12] | 5.53 [4.36;7.07] | 0.359 |
| HGB | 129 [126;141] | 129 [121;142] | 128 [121;138] | 0.539 |

| Characteristics | TACE PVTT=0  N=3073 | Low risk TACE PVTT=1  N=252 | P value |
| --- | --- | --- | --- |
| Gender: |  |  | 0.071 |
| Female | 449 (14.6%) | 48 (19.0%) |  |
| Male | 2624 (85.4%) | 204 (81.0%) |  |
| Age |  |  | 0.641 |
| <60 | 1764 (57.4%) | 149 (59.1%) |  |
| ≥60 | 1309 (42.6%) | 103 (40.9%) |  |
| Etiology: |  |  | 0.19 |
| HBV | 2711 (88.2%) | 226 (89.7%) |  |
| HCV | 68 (2.21%) | 1 (0.40%) |  |
| HBV+HCV | 19 (0.62%) | 2 (0.79%) |  |
| other | 275 (8.95%) | 23 (9.13%) |  |
| ECOG: |  |  | < 0.001 |
| 0 | 2064 (67.2%) | 130 (51.6%) |  |
| 1 | 1000 (32.5%) | 121 (48.0%) |  |
| 2 | 9 (0.29%) | 1 (0.40%) |  |
| Tumor number: |  |  | 0.023 |
| 1 | 1575 (51.3%) | 128 (50.8%) |  |
| 2 | 783 (25.5%) | 49 (19.4%) |  |
| ≥3 | 715 (23.3%) | 75 (29.8%) |  |
| Tumor size | 6.10 [3.80;9.80] | 8.50 [5.30;10.7] | < 0.001 |
| AFP: |  |  | 0.002 |
| <400 | 1940 (63.1%) | 134 (53.2%) |  |
| ≥400 | 1133 (36.9%) | 118 (46.8%) |  |
| ALB | 39.1 [35.6;43.0] | 39.5 [37.2;43.0] | 0.041 |
| TBIL | 16.3 [11.9;23.0] | 15.6 [11.9;19.3] | 0.01 |
| AST | 45.0 [31.0;68.0] | 46.6 [35.0;60.2] | 0.63 |
| ALT | 40.0 [26.0;62.0] | 35.0 [25.0;50.2] | 0.008 |
| PLT | 130 [85.0;184] | 133 [94.0;202] | 0.065 |
| INR | 1.06 [1.00;1.15] | 1.04 [0.97;1.12] | < 0.001 |
| BUN | 5.30 [4.24;6.40] | 4.60 [3.90;6.50] | < 0.001 |
| Cr | 72.0 [62.0;83.0] | 70.0 [58.0;80.0] | 0.036 |
| WBC | 5.24 [4.00;6.86] | 5.13 [4.52;6.98] | 0.651 |
| HGB | 135 [122;148] | 129 [126;141] | 0.011 |

Table S2: Pre-PSM Comparison of TACE without PVTT and low risk PVTT1 underwent TACE

| Characteristics | TACE PVTT=0  N=252 | Low risk TACE PVTT=1  N=252 | P value |
| --- | --- | --- | --- |
| Gender: |  |  | 0.234 |
| Female | 37 (14.7%) | 48 (19.0%) |  |
| Male | 215 (85.3%) | 204 (81.0%) |  |
| Age |  |  | 0.652 |
| <60 | 143 (56.7%) | 149 (59.1%) |  |
| ≥60 | 109 (43.3%) | 103 (40.9%) |  |
| Etiology: |  |  | 0.237 |
| HBV | 226 (89.7%) | 226 (89.7%) |  |
| HCV | 6 (2.38%) | 1 (0.40%) |  |
| HBV+HCV | 1 (0.40%) | 2 (0.79%) |  |
| other | 19 (7.54%) | 23 (9.13%) |  |
| ECOG: |  |  | 0.204 |
| 0 | 147 (58.3%) | 130 (51.6%) |  |
| 1 | 103 (40.9%) | 121 (48.0%) |  |
| 2 | 2 (0.80%) | 1 (0.40%) |  |
| Tumor number: |  |  | 0.984 |
| 1 | 130 (51.6%) | 128 (50.8%) |  |
| 2 | 48 (19.0%) | 49 (19.4%) |  |
| ≥3 | 74 (29.4%) | 75 (29.8%) |  |
| Tumor size | 8.35 [5.38;10.7] | 8.50 [5.30;10.7] | 0.955 |
| AFP: |  |  | 0.789 |
| <400 | 138 (54.8%) | 134 (53.2%) |  |
| ≥400 | 114 (45.2%) | 118 (46.8%) |  |
| ALB | 39.2 [36.1;42.2] | 39.5 [37.2;43.0] | 0.066 |
| TBIL | 16.4 [11.9;23.9] | 15.6 [11.9;19.3] | 0.025 |
| AST | 49.5 [34.9;75.2] | 46.6 [35.0;60.2] | 0.097 |
| ALT | 41.9 [28.0;68.2] | 35.0 [25.0;50.2] | 0.005 |
| PLT | 138 [90.0;195] | 133 [94.0;202] | 0.963 |
| INR | 1.06 [1.00;1.13] | 1.04 [0.97;1.12] | 0.019 |
| BUN | 5.26 [4.37;6.38] | 4.60 [3.90;6.50] | 0.002 |
| Cr | 70.8 [61.0;80.0] | 70.0 [58.0;80.0] | 0.531 |
| WBC | 5.40 [4.19;6.74] | 5.13 [4.52;6.98] | 0.711 |
| HGB | 136 [123;148] | 129 [126;141] | 0.033 |

Table S3: Post-PSM Comparison of TACE without PVTT and low risk PVTT1 underwent TACE

TableS4: Pre-PSM Comparison of systematic therapy and TACE with PVTT1

| Characteristics | systematic therapy  N=167 | Low risk TACE  N=252 | P value |
| --- | --- | --- | --- |
| Gender: |  |  | 0.533 |
| Female | 27 (16.2%) | 48 (19.0%) |  |
| Male | 140 (83.8%) | 204 (81.0%) |  |
| Age |  |  | 0.002 |
| <60 | 124 (74.3%) | 149 (59.1%) |  |
| ≥60 | 43 (25.7%) | 103 (40.9%) |  |
| Etiology: |  |  | 0.202 |
| HBV | 154 (92.2%) | 226 (89.7%) |  |
| HCV | 3 (1.80%) | 1 (0.40%) |  |
| HBV+HCV | 0 (0.00%) | 2 (0.79%) |  |
| other | 10 (5.99%) | 23 (9.13%) |  |
| ECOG: |  |  | < 0.001 |
| 0 | 49 (29.3%) | 130 (51.6%) |  |
| 1 | 118 (70.7%) | 121 (48.0%) |  |
| 2 | 0 (0.00%) | 1 (0.40%) |  |
| Tumor number: |  |  | 0.036 |
| 1 | 98 (58.7%) | 128 (50.8%) |  |
| 2 | 38 (22.8%) | 49 (19.4%) |  |
| ≥3 | 31 (18.6%) | 75 (29.8%) |  |
| Tumor size | 11.0 [8.45;13.9] | 8.50 [5.30;10.7] | < 0.001 |
| AFP |  |  | < 0.001 |
| <400 | 58 (34.7%) | 134 (53.2%) |  |
| ≥400 | 109 (65.3%) | 118 (46.8%) |  |
| ALB | 39.2 [36.0;42.0] | 39.5 [37.2;43.0] | 0.18 |
| TBIL | 18.4 [12.4;24.0] | 15.6 [11.9;19.3] | 0.002 |
| AST | 63.0 [46.5;93.0] | 46.6 [35.0;60.2] | < 0.001 |
| ALT | 42.0 [28.0;66.0] | 35.0 [25.0;50.2] | 0.014 |
| PLT | 140 [95.0;207] | 133 [94.0;202] | 0.746 |
| INR | 1.10 [1.02;1.17] | 1.04 [0.97;1.12] | < 0.001 |
| BUN | 4.70 [3.90;5.77] | 4.60 [3.90;6.50] | 0.862 |
| Cr | 80.5 [69.2;93.0] | 70.0 [58.0;80.0] | < 0.001 |
| WBC | 5.43 [4.25;6.82] | 5.13 [4.52;6.98] | 0.647 |
| HGB | 139 [122;154] | 129 [126;141] | < 0.001 |

| Characteristics | Low risk TACE PVTT=1  N=111 | Sys PVTT=1  N=111 | P value |
| --- | --- | --- | --- |
| Gender: |  |  | 0.324 |
| Female | 27 (24.3%) | 20 (18.0%) |  |
| Male | 84 (75.7%) | 91 (82.0%) |  |
| Age: |  |  | 0.091 |
| <60 | 66 (59.5%) | 79 (71.2%) |  |
| ≥60 | 45 (40.5%) | 32 (28.8%) |  |
| Etiology: |  |  | 0.218 |
| HBV | 103 (92.8%) | 98 (88.3%) |  |
| HCV | 0 (0.00%) | 3 (2.70%) |  |
| other | 8 (7.20%) | 10 (9.00%) |  |
| ECOG: |  |  | 0.253 |
| 0 | 43 (38.7%) | 34 (30.6%) |  |
| 1 | 67 (60.4%) | 77 (69.4%) |  |
| 2 | 1 (0.90%) | 0 (0.00%) |  |
| Tumor number: |  |  | 0.303 |
| 1 | 30 (27.0%) | 25 (22.5%) |  |
| 2 | 63 (56.8%) | 59 (53.2%) |  |
| ≥3 | 18 (16.2%) | 27 (24.3%) |  |
| Tumor size | 10.0 [7.95;11.4] | 9.60 [7.45;11.7] | 0.684 |
| AFP: |  |  | 0.685 |
| <400 | 47 (42.3%) | 51 (45.9%) |  |
| ≥400 | 64 (57.7%) | 60 (54.1%) |  |
| ALB | 39.9 [37.3;43.2] | 39.7 [37.1;42.2] | 0.556 |
| TBIL | 15.0 [12.0;19.0] | 18.5 [12.9;24.0] | 0.002 |
| AST | 47.0 [36.0;63.8] | 60.0 [42.0;82.5] | 0.003 |
| ALT | 34.0 [24.0;47.0] | 44.0 [28.5;67.0] | 0.003 |
| PLT | 141 [97.5;217] | 119 [85.0;169] | 0.019 |
| INR | 1.03 [0.96;1.10] | 1.07 [1.00;1.14] | 0.123 |
| BUN | 4.30 [3.80;6.00] | 4.80 [4.00;5.80] | 0.484 |
| Cr | 68.4 [52.2;78.5] | 71.5 [56.2;79.0] | 0.241 |
| WBC | 5.13 [4.44;6.88] | 5.12 [4.18;6.25] | 0.552 |
| HGB | 130 [126;141] | 132 [120;147] | 0.865 |

Table S5: Post-PSM Comparison of systematic therapy and low risk TACE with PVTT1

Table S6: Multivariable Cox analysis of TACE PVTT0 with low risk TACE PVTT1 after PSM

| Characteristics | coefficient | HR | Lower 95%CI | Upper 95%CI | P value |
| --- | --- | --- | --- | --- | --- |
| PVTT (0 as ref) | -0.0960 | 0.9085 | 0.7200 | 1.146 | 0.4187 |
| ECOG (0 as ref) |  |  |  |  |  |
| 1 | 0.1451 | 1.1561 | 0.9159 | 1.4590 | 0.2221 |
| 2 | 1.1750 | 3.2381 | 1.0208 | 10.272 | 0.0460 |
| Tumor number (1 as ref) |  |  |  |  |  |
| 2 | 0.0332 | 1.0337 | 0.7598 | 1.406 | 0.8328 |
| ≥3 | 0.0039 | 1.0039 | 0.7682 | 1.312 | 0.9775 |
| Tumor size | 0.0290 | 1.0295 | 0.9946 | 1.065 | 0.0985 |
| AFP (<400 as ref) | 0.15751 | 1.1706 | 0.9251 | 1.481 | 0.1897 |
| INR | 0.05621 | 1.0578 | 0.3714 | 3.013 | 0.9162 |

| Characteristics | Coefficient | HR | Lower 95%CI | Upper 95%CI | P value |
| --- | --- | --- | --- | --- | --- |
| Therapy (TACE as ref) | 2.0217 | 7.5513 | 3.4309 | 16.6200 | <0.0001 |
| ECOG (0 as ref) |  |  |  |  |  |
| 1 | -0.5817 | 0.5590 | 0.2529 | 1.2357 | 0.1507 |
| 2 | 0.2392 | 1.2703 | 0.1289 | 12.515 | 0.8376 |
| Tumor size | 0.00812 | 1.0085 | 0.8444 | 1.2036 | 0.9285 |
| ALT | -0.0107 | 0.9893 | 0.9788 | 0.9999 | 0.0481 |

Table S7: Multivariable COX analysis of TACE and systematic therapy with PVTT1 after PSM.
